# Barriers to implementing the WHO Safe Childbirth Checklist in maternity hospitals, Brazil

**DOI:** 10.11606/s1518-8787.2025059006897

**Published:** 2025-12-08

**Authors:** Ewerton William Gomes Brito, Tatyana Maria Silva de Souza Rosendo, Fernanda Pereira Marinho Amaro, Wilton Rodrigues Medeiros, Flôrismeiire de Souza Silva, Marise Reis de Freitas, Rafhael Brito de Almeida Santos, Rose L. Molina, Katherine E. A. Semrau, Lauren Bobanski, Danielle E. Tuller, Zenewton André da Silva Gama

**Affiliations:** I Universidade Federal do Rio Grande do Norte. Departamento de Saúde Coletiva. Natal, RN, Brasil; II Universidade Federal do Rio Grande do Norte. Programa de Pós-Graduação em Gestão da Qualidade em Serviços de Saúde. Natal, RN, Brasil; III Universidade Federal do Rio Grande do Norte. Centro de Ciências da Saúde. Natal, RN, Brasil; IV Universidade Federal do Rio Grande do Norte. Departamento de Infectologia. Natal, RN, Brasil; V Universidade Federal do Rio Grande do Norte. Maternidade Escola Januário Cicco. Natal, RN, Brasil; VI Brigham and Women’s Hospital and Harvard T.H. Chan School of Public Health. Boston, MA, United States; VII Beth Israel Deaconess Medical Center. Department of Obstetrics & Gynecology. Boston, MA, United States

**Keywords:** Checklist, Maternal Health, Health Care Quality, Patient Safety, Implementation Science

## Abstract

**OBJECTIVE:**

To identify barriers to the implementation of the World Health Organization Safe Childbirth Checklist in two reference maternity hospitals—one for high-risk and one for routine-risk childbirths—and to develop a causal model applicable to these contexts.

**METHODS:**

This qualitative, exploratory study was conducted in two public maternity hospitals that had been using the checklist, since its implementation in 2014. Data were collected through focus groups interviews and brainstorming sessions conducted in 2022 and 2023. Participants included healthcare professionals involved in childbirth care and members of the patient safety center. Content analysis categorized findings based on the five domains of the Consolidated Framework for Implementation Research (CFIR). A causal model was developed using a fishbone diagram to organize results by category.

**RESULTS:**

The identified barriers were classified into four of the five CFIR domains. In the Innovation domain, the checklist itself posed challenges due to its design, complexity, and adaptability to existing workflows. In the Inner Setting, barriers included a weak patient safety culture and infrastructure limitations. The Implementation Process domain revealed deficiencies such as inadequate planning, lack of stakeholder involvement, and absence of feedback and assessment mechanisms. Unlike the high-risk maternity hospital, the Outer Setting barrier —lack of policies supporting continuing education—was identified in the routine-risk facility.

**CONCLUSIONS:**

Implementation of the checklist in the studied maternity hospitals is hindered by structural, cultural, and adaptation challenges. Limited investment in training represents a significant obstacle, highlighting the need for professional development programs. High staff turnover and the absence of specific protocols further compromise consistent use. Addressing these barriers requires comprehensive strategies to enhance adherence to and integration of the checklist in maternal-newborn care.

## INTRODUCTION

Maternal and neonatal mortality remain a significant global health challenge, despite advances in recent decades. The latest estimates indicate approximately 287,000 maternal deaths, 1.9 million stillbirths, and 2.3 million neonatal deaths worldwide^1,2^. Brazil has a high maternal mortality ratio (68.0 per 100,000 live births), particularly among Black women, and continues to struggle to reduce neonatal mortality^3^. Most of these deaths are attributable to preventable causes, demonstrating the need to improve the quality of childbirth care and to address structural racism^4^.

The World Health Organization (WHO) Safe Childbirth Checklist (SCC) is a tool designed to improve childbirth care quality and promote safe practices. It addresses key causes of maternal deaths (hemorrhage, hypertensive disorders, infection), fetal deaths, and neonatal deaths (asphyxia, infection, prematurity). By fostering adherence to evidence-based practices, the SCC helps healthcare professionals optimize workflows and reduce adverse outcomes during childbirth^5^.

Recent evidence highlights the positive impact of the SCC on essential practices, such as managing pre-eclampsia, preventing infections, using the partogram, postpartum counseling, reducing stillbirths, and minimizing errors caused by workload^6,7^. In Brazil, studies have shown increased adherence to critical practices following SCC implementation. Sousa et al.^8^ reported improvements in partogram use, oxytocin administration, timely umbilical cord clamping, and skin-to-skin contact. Similarly, Gama et al.^9^ observed better adherence to magnesium sulfate protocols for managing pre-eclampsia and severe pre-eclampsia.

Adherence to the SCC remains challenging despite its benefits. Praxedes et al.^10^ reported that in a maternity hospital in Northeastern Brazil, only 71% of medical records included the checklist, with an average of 24% of items completed and only 0.1% fully completed. However, continued implementation efforts led to improved adherence and reduced adverse events^8^. Adherence varies across pause points, as shown in a Tanzanian study in which completion rates were highest at discharge (93%) and lowest during delivery (39%)^11^. Sustained SCC use relies on strategies such as team coaching, motivation, monitoring, feedback, and problem-solving^12^.

Understanding the factors that hinder or facilitate the implementation and dissemination of new patient safety tools is essential^13^. An integrative review identified several barriers to SCC use, including cultural (interpersonal relationships, professional hierarchy, and poor communication), structural (design and font used in the SCC), and work process-related factors (implementation design, management attitudes, and training needs for healthcare professionals)^14^. However, these findings fail to consider the differences across maternity contexts, such as large, high-risk referral hospitals versus smaller, routine-risk facilities.

Implementation science frameworks are valuable for systematizing barriers and guiding the design of interventions to accelerate WHO SCC implementation^13^. Determinant frameworks, such as the updated Consolidated Framework for Implementation Research (CFIR)^15^, are recommended to identify and classify barriers. These barriers can be organized into an improvement theory using logic models, such as fishbone diagrams, which help illustrate causal relationships and accelerate progress in this area^16^.

A study in Mozambique using the CFIR framework identified barriers to SCC use, including underfunded maternal care, insufficient infrastructure and human resources, and low provider motivation^17^. Evaluations of SCC implementation in diverse regions (Africa, Southeast Asia, Europe, North America) highlight the importance of local adaptation and the use of an implementation guide for sustainability^18^. This study employed CFIR to identify barriers in maternity hospitals already using the SCC, contrasting with the Mozambique study^17^, which focused on pre-implementation barriers.

Given the need for empirical research to promote the dissemination of the SCC in Brazil and, consequently, the implementation of safe childbirth practices, this study aims to identify barriers to the use of the WHO SCC in two Brazilian maternity hospitals with different risk profiles. To facilitate replication in different settings and enable synthesis in future reviews, these barriers were identified and analyzed using the updated CFIR framework.

## METHODS

### Study Design

This qualitative study aims to identify barriers to SCC adherence in two maternity hospitals. The research analyzed barriers and facilitators, which is a strategic approach in dissemination and implementation studies in health^15^. A deductive qualitative approach was used, with interview content categorized according to the updated CFIR framework.

### Study Context

This study is part of a multicenter, mixed-methods project designed to implement a multifaceted, SCC-centered strategy to improve childbirth quality and safety. The project involves six Brazilian maternity hospitals—five in the state of Rio Grande do Norte and one in the state of Rio de Janeiro—and is a collaboration between the Quality in Health Services research group (QualiSaúde) at Universidade Federal do Rio Grande do Norte (UFRN) and the Laboratory of Quality and Patient Safety at Universidade Federal Fluminense. Approved for 2022–2025, the project is funded by the Conselho Nacional de Desenvolvimento Científico e Tecnológico.

The study was conducted in two public maternity hospitals in Rio Grande do Norte State, both affiliated with UFRN, selected for their long-standing use of the SCC since 2014 and their distinct institutional profiles. One hospital (M1), a teaching facility in the state capital, specializes in high-risk pregnancies and performs about 4,000 deliveries annually. The other (M2), located in the interior of the state, manages routine-risk pregnancies and conducts approximately 2,300 deliveries annually. Both hospitals have quality management departments with patient safety centers (PSCs) supporting SCC implementation.

### Study Participants

The professionals selected to report on barriers and facilitators had prior experience using or promoting the SCC at the hospital. Convenience sampling was applied, targeting professionals directly involved with the SCC, either in quality management or maternal-newborn care. Participants included professionals from the PSCs and the hospital infection control committee, obstetricians, pediatricians, pediatric and gynecology/obstetrics residents, nurses, and nursing technicians. Exclusion criteria were professionals not on duty during data collection or those engaged in patient care at that time.

### Data Collection

Two focus groups interviews were conducted, one at each maternity hospital, in December 2022 and May 2023. A total of 15 professionals participated at the routine-risk maternity hospital (M2), and nine at the high-risk maternity hospital (M1). Participants were invited by hospital management using purposive sampling based on their direct experience with SCC implementation and use. Each session lasted approximately 90 minutes.

At the beginning of each session, the researchers explained the purpose of the activity, providing context about the project and the involvement of UFRN. After a brief explanation of the method used to diagnose barriers, the issue of low SCC use was written on a whiteboard.

Next, a brainstorming session was conducted with the professionals, guided by the question: “What do you identify as a barrier to using the SCC?” Participants were encouraged to list perceived barriers based on their involvement in the care process. Moderators recorded contributions directly on a whiteboard.

The identified barriers were then categorized according to their respective cause groups, following methodological guidelines for creating an Ishikawa diagram—a quality tool that visually represents possible causes leading to a specific outcome or problem.

The Ishikawa diagram, also known as the fishbone or cause-and-effect diagram, is a graphical representation that enables the identification of primary and secondary causes of a problem. This tool is easy to apply, albeit limited to identifying one problem at a time^19^. The methodological steps recommended by Saturno^20^ were followed: documenting the problem, analyzing cause groups, and identifying first-level, second-level, third-level, and subsequent causes. At each step, the question “Why does this happen?” was presented, and responses were collected using the brainstorming technique.

The focus group interviews were moderated by three members of the research team: two researchers from the QualiSaúde research group at UFRN, both holding master’s degrees in quality management in health services, and one professor with a PhD from the Department of Collective Health at UFRN, also affiliated with QualiSaúde. The sessions were not audio-recorded or transcribed verbatim. Instead, moderators took detailed notes during the sessions, which were subsequently transcribed into a Word document to support systematic qualitative analysis.

### Data Analysis

The qualitative data collected from the maternity hospitals were analyzed using a pragmatic approach^21^. We conducted a framework analysis, the history, philosophical assumptions, and main processes of which have been described elsewhere^22^. The CFIR was adopted^17^, as it is widely used in implementation science to assess contextual factors and to predict or explain barriers and facilitators affecting implementation effectiveness^17^.

The process was conducted in five stages. First, a group of four researchers was trained by a senior researcher specialized in CFIR application. Subsequently, the trained researchers, all specialized in health services quality management, reviewed the focus group interviews in detail and reached consensus in collaboration with two leaders of SCC implementation. The group then classified the data according to CFIR constructs: innovation characteristics (eight constructs), inner setting (11 constructs), outer setting (seven constructs), individual characteristics (13 constructs), and implementation process (nine constructs). Data classification was subsequently validated by another researcher with extensive CFIR experience. Finally, the trained researchers reviewed and compared the identified patterns between the two maternity hospitals and interpreted the data to analyze barriers to SCC use.

### Ethical Considerations

The project complies with Resolution 466/2012 and was approved by the UFRN Research Ethics Committee of Hospital Universitário Onofre Lopes (CEP-HUOL/UFRN), with protocol number 5.621.794 of 09/02/2022.

## RESULTS

### Participant Characteristics

The study considered the perceptions of professionals directly involved with SCC implementation. At M1 (high risk), participants included physicians, nurses, residents, and members of the PSC and the hospital infection control committee. At M2 (routine risk), participants included nurses, nursing technicians, residents, a public health specialist, a unit chief, and administrative support staff.

### Innovation

At M1, the SCC’s design and overlap with other mandatory forms were identified as key factors contributing to resistance. The complexity, redundancy, and suboptimal layout of the checklist make its completion difficult in high-pressure contexts with heavy clinical demand.

At M2, barriers related to the adaptability of the checklist were more prominent. The SCC content does not fully reflect local clinical protocols or available resources, particularly in the surgical center, where its structure is less compatible with workflow demands.

### Inner Setting

Both hospitals reported significant structural barriers, although with some differences.

At M1, high turnover of students and healthcare professionals was reported as a key factor affecting continuity of SCC use. High workload and an excessive number of forms to be completed further reduced adherence. These barriers are compounded by perceptions that the SCC duplicates other documentation.

At M2, the hospital is undergoing a transition toward higher care complexity, with increasing patient volumes and acuity. This has led to greater fragmentation of the care process across professional groups, undermining a shared sense of responsibility for SCC use. The structure of the checklist—spanning different care moments—also contributes to low adherence.

Barriers related to organizational culture were also reported in both hospitals. At M1, a low level of staff awareness regarding patient safety was described. At M2, knowledge of patient safety and the importance of the SCC remains limited, particularly among residents and newly hired staff.

Regarding “access to knowledge and information,” M1 participants reported a lack of structured continuing education regarding the SCC. No formal updates on SCC use have been provided since the initial implementation phase.

In relation to “compatibility,” M1 participants noted that SCC completion is not well integrated into medical records or discharge routines, contributing to inconsistent use. The checklist is not readily available at the point of discharge, and professionals perceive its completion as redundant in relation to existing forms.

### Implementation Process

In both hospitals, a lack of clarity regarding who is responsible for completing the SCC was reported, leading to variability in adherence. At M2, previous efforts to digitize the SCC were not sustained, creating confusion among staff about whether to complete the checklist on paper or electronically.

At M1, the absence of regular feedback on SCC use and related care outcomes was highlighted as a barrier to team engagement.

### Outer Setting

At M2, institutional regulations limiting staff participation in training activities during working hours were identified as an important barrier. This constraint has limited opportunities to strengthen knowledge and motivation related to SCC use.

The identified barriers are summarized in Box. To facilitate visualization, a cause-and-effect diagram integrating findings from both maternity hospitals was developed and is presented in Figure.

**Box:** Barriers to adherence to the safe childbirth checklist in two maternity hospitals according to the Consolidated Framework for Implementation Research.

Innovation	
Complexity of the innovation (M1)	Unnecessary items (M1)
Design of the innovation (M1)	Repeated items (M1) Poor layout (M1)
Adaptability of the innovation (M2)	Lack of items that account for other therapeutic decisions (M2) Difficult usability in cesarean sections (M2)
Outer setting	
Policies and laws (M2)	Institution guidelines that limit continuing education activities for the SCC (M2)
Inner setting	
Structural characteristics – Work infrastructure (M1, M2)	Student and staff turnover (M1) High work demand (M1) and (M2) Excessive number of forms to fill out, especially at discharge (M1) Fragmentation of work processes across professional categories (M2) Rising birth volume and care complexity hindering SCC use (M2)
Culture – Focus on learning (M1, M2)	Low sensitivity to the topic (M1) Poor knowledge of patient safety and the SCC in residents and staff (M2)
Compatibility (M1)	Items duplicated in relation to other mandatory forms (M1) SCC not readily available at discharge – Organization of medical records (M1) Fragmented care (M1)
Access to knowledge and information (M1)	Lack of continuing education for medical teams (M1)
Implementation process	
Planning (M1, M2)	Responsibility for form completion undefined – Lack of a protocol (M1) Unclear whether to complete forms digitally or on paper (M2)
Engaging (M1)	Lack of SCC training during onboarding/orientation of students and staff (M1) Lack of feedback on care outcomes (M1)
Reflecting and evaluating (M1)	

M1: maternity 1; M2: maternity 2; SCC: Safe Childbirth Checklist.

**Figure:**
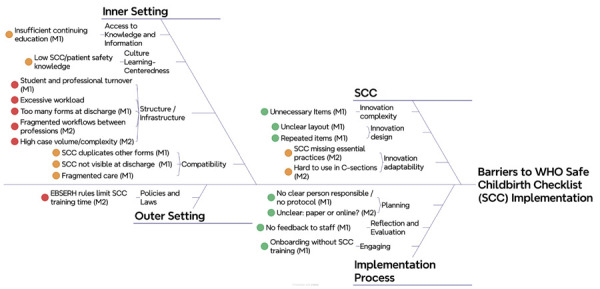
Cause-and-effect diagram. High-risk maternity hospital (M1) and routine risk maternity hospital (M2). Brazil, 2023.

Note: barrier classification for implementing the World Health Organization Safe Childbirth Checklist: green – easily modifiable by local managers; orange – requires moderate effort; red – difficult to modify.

## DISCUSSION

Use of the CFIR enabled a systematic analysis of primary barriers to SCC adherence in two pioneering maternity hospitals in Brazil that have implemented the tool. Thus, this study can help other institutions anticipate and address key barriers in their implementation processes.

Understanding the causes behind incomplete checklist completion enables targeted interventions to improve adherence. This contributes to safe practices and quality care, as the SCC was designed to achieve this goal and evidence shows its effects in increasing the implementation of best practices^6,8^. Rational SCC use aligns with the principles of the Brazilian Unified Health System (SUS), known for its universality and comprehensiveness, which organizes maternal-child healthcare via the *Rede Cegonha* (Stork Network), recently renamed Rede Alyne. One of its guidelines is to ensure best practices and safety in childbirth. Thus, this innovation could contribute to improving maternal-child care in Brazil.

Moreover, the barriers identified in this study—such as high staff turnover, low safety culture, and lack of standardized implementation protocols—reflect structural challenges commonly faced in SUS maternity hospitals. These findings provide practical insights for tailoring continuing education policies, reinforcing patient safety centers, and integrating tools such as SCC into national maternal and child health strategies. By aligning implementation efforts with SUS principles and policy instruments, including national quality improvement programs and interprofessional training initiatives, the SCC can be more effectively incorporated into routine practice across diverse care settings, thereby strengthening equity and safety in childbirth care nationwide.

Our findings reinforce previous research indicating that SCC implementation is not merely a technical process but is strongly shaped by contextual, cultural, and organizational factors. Similar to a study conducted in Mozambique, the barriers identified were linked to local challenges, including insufficient material and human resources, inadequate training, perceptions of increased workload associated with SCC use, low staff motivation, and underfunded maternal-child health care^17^. Our study contributes to this literature by focusing on barriers to sustaining SCC use beyond its initial implementation.

In both maternity hospitals, we found that barriers related to the innovation itself —including redundant items, poor layout, and difficulties in adapting the checklist to local protocols—interact with structural challenges such as high staff turnover and fragmented work processes. Notably, when the SCC was adapted for the hospitals studied, a participatory approach was adopted, involving several consensus techniques, expert validation, and staff training^23^, following WHO guidelines^24^. However, structural and personnel changes over time appear to have undermined this initial integration, underscoring the need for ongoing review and adaptation of the tool.

There is an opportunity to better integrate the checklist as an instrument to strengthen service delivery and promote its routine use as a health protection tool that may reduce care risks. A 2014 study conducted at the same high-risk maternity hospital, shortly after the checklist’s implementation, found barriers to adherence such as cultural resistance, limited understanding of its purpose, and difficulties among professionals in recognizing its benefits^10^.

A particularly salient finding was the contrast in organizational culture between both hospitals. In M2, discussions indicated a more favorable context for SCC implementation, possibly reflecting a stronger safety culture. Conversely, in M1, high clinical demand, high staff and student turnover, and a weak learning culture contributed to inconsistent SCC use. Research assessing SCC in maternity hospitals remains scarce, highlighting this as a little-explored area. One of the few studies found, assessing three Brazilian maternity hospitals, did not find strong safety culture dimensions in any of them^25^.

Within safety culture, professionals mentioned fragmented work and lack of communication, especially between multidisciplinary teams and the medical staff. Albolino et al.^26^ found high adherence to the SCC among obstetric nurses and midwives (96%) but very low adherence among obstetricians (3%). This suggests that while the checklist aims to promote interdisciplinary work, field studies generally reveal poor communication across teams and strong hierarchical dynamics rather than partnerships. Additionally, overconfidence among tertiary-level professionals may contribute to underestimating the checklist’s importance^10,27^.

Workload and infrastructure constraints, particularly patient overcrowding in M1 and increasing care complexity in M2, were also highlighted as important contextual factors. The high-risk maternity ward deals with overcrowding almost daily, serving as an entry point for gynecological and obstetric emergencies and handling a significant portion of high-risk pregnancies in the state. In a qualitative study, Kouroma et al.^28^ underscored the challenges of using the SCC when managing a large number of patients simultaneously. Similarly, the low-risk maternity hospital has experienced increased care complexity due to changes in its service profile, compounded by the absence of a protocol that defines responsibilities for completing the checklist.

Overall, many of the barriers identified can be traced to gaps in the implementation process. Following SCC adoption, it is crucial to develop and maintain a strategic plan that clearly defines who is responsible for checklist completion, integrates SCC use into routine workflows, and provides ongoing staff education and support. Studies indicate that SCC implementation is more effective when embedded in broader quality improvement initiatives and supported by leadership, monitoring, and coaching^18,29^.

Recent research shows that checklists can improve healthcare services and increase adherence to best practices. The SCC, as a relatively low-cost tool, can and should be adapted to different contexts^24^. However, it does not generate significant change on its own; it is more effective when implemented within broader quality improvement initiatives^30^. Given these findings, it is essential to strengthen the implementation context by fostering safety culture assessments, reinforcing patient safety centers, supporting leadership, and building quality improvement infrastructure. Additionally, it is important to implement monitoring with clear indicators and provide feedback to professionals.

This qualitative study was conducted in two pioneering Brazilian maternity hospitals implementing the SCC, the only ones included in national studies to date. It aimed to generate hypotheses and gather management-relevant information rather than make generalizations. However, the choice of university maternity hospitals may limit its external validity, as the barriers identified might be specific to these settings.

Participants were selected based on availability and interest, and leadership involvement in focus groups may have introduced bias. Engaged participants may have differing opinions from non-participants. Moreover, data collection relied on brainstorming and discussions, which are subject to recall bias or the tendency to present experiences in a favorable or unfavorable light. Despite these limitations, the innovative application of the CFIR in maternity hospitals already using the SCC enabled the identification of barriers to its sustainable use.

## CONCLUSIONS

Implementation of the SCC in Brazilian maternity hospitals faces multiple challenges, including structural, cultural, design, and adaptation issues. Significant obstacles include limited investment in continuing education and SCC training, underscoring the urgent need for structured professional development programs to improve adherence and understanding of the importance of this tool for maternal-newborn safety. Additionally, high staff turnover and the lack of clear protocols for completing the SCC can compromise its use. To ensure SCC adherence, it is essential for maternity hospitals to address specific issues by implementing strategies that promote safety culture assessments, reinforce interprofessional education among residents, strengthen patient safety culture, and develop sustainability strategies for the continued use of the SCC.

## Data Availability

The research data are available from the corresponding author upon reasonable request.
